# Interday variation and effect of transportation on indirect blood pressure measurements, plasma endothelin-1 and serum cortisol in Standardbred and Icelandic horses

**DOI:** 10.1186/1751-0147-54-37

**Published:** 2012-06-10

**Authors:** Josefin Söder, Johan T Bröjer, Katarina EA Nostell

**Affiliations:** 1 Department of Clinical Sciences, Swedish University of Agricultural Sciences, Box 7054, Uppsala, S-750 07, Sweden

**Keywords:** Horse, Plasma endothelin-1, Cortisol, Blood pressure, Transportation

## Abstract

**Background:**

Systemic hypertension is a prominent feature in humans with metabolic syndrome (MS) and this is partly caused by an enhanced endothelin-1 (ET-1) mediated vasoconstriction. There are indications that systemic hypertension might be a feature in equine metabolic syndrome (EMS) but if ET-1 is involved in the development of hypertension in horses is not known. Increased levels of cortisol have also been found in humans with MS but there are no reports of this in horses. Before blood pressure, plasma ET-1 and serum cortisol can be evaluated in horses with EMS, it is necessary to investigate the interday variation of these parameters on clinically healthy horses. The aims of the present study were therefore to evaluate the interday variation and influence of transportation on systemic blood pressure, plasma ET-1 and serum cortisol in healthy Standardbred and Icelandic horses, and to detect potential breed differences.

**Methods:**

Nine horses of each breed were included in the study. Blood pressure was measured and blood samples were collected between 6 and 9 am on two separate days. Eight of the horses (four of each breed) were transported to a new stable were they stayed overnight. The next morning, the sampling procedure was repeated.

**Results:**

The interday variation was higher for plasma ET-1 (37%) than for indirect pressure measurements (8-21%) and serum cortisol (18%). There were no differences in systemic blood pressure between the two breeds. The Icelandic horses had significantly lower serum cortisol and significantly higher plasma ET-1 concentrations compared to the Standardbred horses. Plasma ET-1 was significantly elevated after transportation, but systemic blood pressure and serum cortisol did not differ from the values obtained in the home environment.

**Conclusions:**

Indirect blood pressure, plasma ET-1 and serum cortisol are of interest as markers for cardiovascular dysfunction in horses with EMS. The elevated plasma ET-1 concentrations recorded after transportation was likely caused by a stress response. This needs to be considered when evaluating plasma ET-1 in horses after transportation. The differences detected in plasma ET-1 and serum cortisol between the two breeds might be related to differences in genetic setup, training status as well as management conditions.

## Introduction

Equine metabolic syndrome (EMS) was introduced in veterinary medicine in 2002 and is a clinical condition characterized by obesity, regional adiposity, insulin resistance (IR) and clinical or subclinical laminitis [1,2]. A similar syndrome exists in human medicine; the metabolic syndrome (MS), which is characterized by IR, abdominal obesity, dyslipemia, coagulopathies, hyperglycemia and systemic hypertension [3]. There are indications that systemic hypertension might be a feature in EMS, as ponies with EMS and a previous history laminitis have been reported to have elevated systemic blood pressures during summer [4]. The systemic hypertension seen in obese MS patients with IR is partly due to a vascular endothelial dysfunction with a decreased production of the vasodilator nitric oxide and an enhanced endothelin (ET-1) mediated vasoconstriction [5-7]. If ET-1 is involved in the development of the previously reported seasonal hypertension in ponies is not known, as there are no studies that describe plasma ET-1 levels in horses diagnosed with EMS.

In humans, the similarities between MS and Cushings´s disease has raised the question if elevated cortisol levels are associated with the development MS [8,9]. There are studies that have shown that patients with MS have higher circulating concentrations of cortisol, but there are also studies that have failed to find this relationship [10,11]. To our knowledge there are no studies that have measured serum cortisol concentrations in horses diagnosed with EMS, and the question remains whether horses with EMS differ in cortisol concentration compared to controls. Cortisol concentrations have a circadian rhythm in horses but this rhythm is easily disturbed by factors such as transportation and changes in management and feeding [12,13]. The diagnostic test often used to detect horses suffering from EMS is a combined glucose and insulin tolerance test (CIGT-test) [2,14] and the test is usually performed in a clinical setting. The stress response caused by the transportation to the clinic and the change in environment has the potential to affect not only cortisol concentrations, but also the results of other markers for metabolic and cardiovascular changes used for diagnosing the disease. Before blood pressure, plasma ET-1 and serum cortisol can be evaluated in horses with EMS, it is therefore necessary to investigate the interday variation of these parameters on clinically healthy horses.

The aims of the present study were therefore to evaluate the interday variation and influence of transportation on systemic blood pressure, plasma ET-1 and serum cortisol in healthy Standardbred and Icelandic horses, and to detect potential breed differences. An additional aim was to compare two different oscillometeric blood pressure measurement devices, the Cardell Veterinary Vital Signs Monitor (Cardell) and the Memo Diagnostic High Definition Oscillometry Monitor (HDO).

## Materials and methods

The study was sanctioned (C27/11) by the Ethical Committee for Animal Experiments, Uppsala, Sweden.

### Horses

Nine Standardbred (7 mares and 2 geldings; body weight 425–586 kg; age 6–20 years) and nine Icelandic horses (7 mares and 2 geldings, body weight 333–444 kg; age 10–18 years) were included in the study. The Standardbred horses were owned by the Department of Clinical Sciences and kept in box stalls with a daily turn out in a paddock. The horses were fed a standardized diet consisting of hay (three times/day) and concentrate (once daily) and provided water ad libitum. The Icelandic horses were privately owned and mainly in free range in a paddock but were also kept in box stalls on a regular basis. These horses were fed grass haylage (three times/day) and provided water ad libitum. All Standardbreds were sedentary horses whereas eight of the Icelandic horses performed lighter exercise three to five times a week and one was sedentary. Four of the Standardbred horses (three mares and one gelding; body weight 425–586 kg; 6–19 years) and four of the Icelandic horses (two mares and two geldings; body weight 326–412 kg; 10–18 years) were included in the transportation study. None of the horses included in the study showed any signs of disease on the clinical examination that was performed before the start of the study.

### Blood pressure measurement devices

Systolic, diastolic, and mean indirect arterial pressures were obtained with Cardell (Cardell Veterinary Monitor 9402, CAS Medical Systems, Brandford, CT) and HDO (Memo Diagnostic High Definition Oscillometry Monitor, horse model) oscillometric monitors. For the transportation study only the HDO device was used. For each monitor, indirect blood pressure was obtained from cuffs placed at the base of the tail (middle coccygeal artery) according the manufacturer’s instructions. The cuff size for the Cardell device was 11 cm and 8 cm for the HDO device. Both devices automatically inflate an occluding cuff and use the oscillometric measurement technique for determination of systolic, diastolic, mean arterial blood pressure and heart rate. The cuffs were repositioned as needed to acquire data acceptable to the internal sensor of the monitor. The same cuff size accompanied each device through all measurements.

### Experimental protocol

The blood pressure measurements and the sample collection were performed between March and April 2011, before the horses had access to summer pasture. The arterial systemic blood pressure was measured between 6 and 9 am on two separate days (M1 and M2). The cuffs were placed on the base of the horse’s tail while the horse was standing in the box stall. Five consecutive determinations of systolic and diastolic blood pressure were performed on each horse, using both devices. On the first day of the study, the starting order in which of the two blood pressure measurement devices were used was randomized by raffle. The next day the starting orders of the devices were switched. In each horse, blood samples were drawn immediately after both blood pressure measurements had been completed.

### Transportation study

The horses were transported in a two-horse trailer for one hour to a new stable were they stayed overnight. The next morning, the blood pressure was measured (HDO) and blood samples collected (M3) in the same manner as previously described.

### Sampling

Blood samples for analysis of plasma ET-1 and serum cortisol were drawn using vacutainer technique and collected in evacuated NaEDTA and serum glass tubes (Vacutainer, BD, Belliver Industrial Estate, Plymouth, UK). In each horse, the blood samples were drawn immediately after the blood pressure measurements had been completed. All samples were kept on ice until centrifuged for 10 min (+4°C, 2700 *g*), plasma and serum subsequently harvested and stored in a −80°C freezer until analysis.

### Sample analysis

Plasma ET-1 concentrations were measured in duplicate with a commercial ELISA-kit (Endothelin (1–21) enzyme immunoassay, Biomedica Medizinprodukte GmbH & Co KG, Wien, Austria). Serum cortisol concentrations (hydrocortisone Compound F) were measured in duplicates with a radioimmunoassay-kit (Coat-A-Count® Cortisol, TKCO2, SIEMENS, USA).

### Statistical methods

In each horse, the lowest and highest recorded value of the five consecutive blood pressure determinations were excluded and the mean systolic and diastolic pressure was calculated from the three remaining determinations (triplicates). The statistical analysis was performed using SigmaPlot software version 11 (SigmaPlot software version 11, SPSS Science, USA).

The results of repeated tests for indirect blood pressure measurement, plasma ET-1 and serum cortisol were compared by use of a paired *t*-test (M1 and M2) or one way repeated measures analyses of variance (M1, M2, and M3; transportation study). Differences between individual means for the three days were tested with a tukey test. Differences in the two breeds for measurement of indirect blood pressure measurement, plasma ET-1 and serum cortisol were compared by use of a *t*-test whereas differences between the blood pressure measurement devices were compared by use of a paired *t*-test. Measurement error, defined as the variation between measurement of the same quantity on the same animal, blood pressure device and ELISA- and radioimmunoassay-kit, was expressed as the coefficient of variation (CV). The standard deviation for duplicate or triplicate measurements was calculated according to Bland [15]. The CV was calculated as the SD divided by the means of each set of two or three measurements and expressed in percentage. Values are expressed as mean ± SD. The level of significance was set at (*P* ≤ 0.05).

## Results

### Systolic and diastolic indirect blood pressure measurements

The mean systolic blood pressure differed significantly between the two devices (HDO, 114 ± 14 mmHg; Cardell, 104 ± 12 mmHg), but not the mean diastolic blood pressure (HDO, 71 ± 11 mmHg; Cardell, 65 ± 13 mmHg) (Table 1). The CV for three repeated measurements on the same occasion were 5.4% in systole and 13.9% in diastole for the Cardell device and 9.5% in systole and 12.1% in diastole for the HDO device. The Cardell showed a better inter-assay CV in systole than the HDO, but there was no difference between the two devices in diastole. Regardless of which blood pressure measurement device that was used, there was no difference in mean systolic or diastolic blood pressure between M1 and M2 (Table 1) or between the two breeds.

**Table 1:** Interday variation in systemic blood pressure (mmHg) and mean concentrations of plasma ET-1 (pg/ml) and serum cortisol (mmol/l)

**Variable**		**M1**	**M2**	**CV %**
**HDO**				
BP_S_ (mmHg)		114 ± 14	113 ± 18	15
BP_D_ (mmHg)		71 ± 11	67 ± 15	21
**Cardell**				
BP_S_ (mmHg)		104 ± 12	102 ± 10	8
BP_D_ (mmHg)		65 ± 13	60 ± 10	19
**ET-1** (pg/ml)		2.3 ± 1.4	2.7 ± 2.0	37
**Cortisol** (nmol/l)		129 ± 76	126 ± 81	18

Mean (±SD) values for systemic, systolic and diastolic blood pressure (measured with the HDO and the Cardell device), plasma ET-1 and serum cortisol concentration measured on two separate days (M1 and M2). No significant differences between M1 and M2 were detected for any of the variables. (*P ≤ 0.05*) *BP*_*S*_: systolic blood pressure, *BP*_*D*_: diastolic blood pressure, *CV*: Coefficient of variation. n = 18, except for plasma ET-1 where n = 16.

### Plasma ET-1 and serum cortisol

One Icelandic horse had plasma ET-1 concentrations that were considered as unreasonably high (16.41 and 14.59 pg/ml) and another Icelandic horse showed a great variation in plasma ET-1 concentrations between measurements (3.09 and 14.54 pg/ml). These values were therefore excluded from the calculations of plasma ET-1. One of these horses took part in the transportation study, and the ET-1 values of this horse were excluded since it had concentrations above the detection limit. There was no significant difference between M1 and M2 in the mean concentration of plasma ET-1 and serum cortisol (Table 1). The intra-assay CV for plasma ET-1 was 6.7% and the intra-assay CV for serum cortisol was 5.7%. Mean concentrations of plasma ET-1 and serum cortisol both differed significantly between the two breeds with the Icelandic horses having significantly higher plasma ET-1 concentrations and significantly lower serum cortisol concentrations compared to the Standardbred horses (Table 2).

**Table 2:** Systolic and diastolic blood pressure (mmHg) and mean concentrations of plasma ET-1 (pg/ml) and serum cortisol (mmol/l) in Standardbred and Icelandic horses

**Variable**		**Standardbred horses**	**Icelandic horses**
**HDO**			
BP_S_ (mmHg)		118 ± 17	110 ± 11
BP_D_ (mmHg)		74 ± 11	67 ± 11
**Cardell**			
BP_S_ (mmHg)		103 ± 9	105 ± 14
BP_D_ (mmHg)		64 ± 7	66 ± 17
**ET-1** (pg/ml)		1.5 ± 0.5	3.2 ± 1.7^*^
**Cortisol** (nmol/l)		188 ± 63	70 ± 22^*^

Mean (±SD) values for systemic, systolic and diastolic blood pressure (measured with the HDO and the Cardell device), plasma ET-1 and serum cortisol concentration measured in two different breeds (Standardbred horses and Icelandic horses). ^*^ P 「 0.05 versus Standardbred horses. *BP*_*S*_: systolic blood pressure, *BP*_*D*_: diastolic blood pressure. n = 9, except for plasma ET-1 in the Icelandic horses where n = 7.

### Influence of transportation

The transportation and new environment did not affect the mean systolic or diastolic blood pressure or mean serum cortisol concentration (Table 3). The mean plasma ET-1 concentration was significantly higher when measured after transportation compared to the home environment (Table 3).

**Table 3:** The effect of transportation and new environment on systemic blood pressure (mmHg) and mean concentrations of plasma ET-1 (pg/ml) and serum cortisol (mmol/l)

**Variable**		**M1**	**M2**	**M3**	**CV %**
**HDO**					
BP_S_ (mmHg)		114 ± 6	110 ± 11	119 ± 10	6
BP_D_ (mmHg)		71 ± 6	67 ± 9	75 ± 7	9
**ET-1** (pg/ml)		1.7 ± 0.9	1.7 ± 0.9	2.6 ± 1.2^*^	31
**Cortisol** (nmol/l)		113 ± 60	115 ± 74	122 ± 35	33

Mean (±SD) values for systemic, systolic and diastolic blood pressure (measured with the HDO), plasma ET-1 and serum cortisol concentration measured on two separate days (M1 and M2) and after transportation (M3). ^*^P 「 0.05 versus MI and M2. *BP*_*S*_: systolic blood pressure, *BP*_*D*_: diastolic blood pressure, *CV*: coefficient of variation. n = 8, except for plasma ET-1 where n = 7.

## Discussion

The results of the present study showed that the interday variation differed between the studied parameters with plasma ET-1 showing the greatest interday variation. The wide range in interday variation is to be expected as similar interday fluctuations have been reported for other hormones and physiological parameters [16,17]. The concentrations of plasma ET-1 measured in the present study was higher for both breeds compared to the concentrations reported previously in healthy Thoroughbred and Standardbred horses [18] but similar to those reported in horses with summer pasture associated recurrent airway obstruction, SPA-RAO [19] and horses with clinical endotoxaemia [20]. The discrepancy in results between studies are likely related to the use of different ELISA assays [19,20], all developed for quantification of human ET-1, as well as genetic differences between breeds. The assay used in the present study had a cross-reactivity with equine ET-1 of a 100%. It is therefore possibly that assays with lower cross-reactivity might underestimate endothelin concentrations.

An interesting finding was the difference in plasma ET-1 and serum cortisol concentrations between the two breeds. The results of the present study showed that the Icelandic horses had significantly higher plasma ET-1 concentrations than the Standardbred horses. There was a considerable individual variation in the concentrations of plasma ET-1 in both breeds, which is in agreement with previous studies in both healthy horses and horses affected of SPA-RAO [19]. However, the individual variation was greater in the Icelandic horses. The reason for this difference between breeds is not known, but might be related to both genetic differences as well differences in management. Previous studies in ponies and Icelandic horses have reported that they are partially insulin resistant compared to normal horses [21-23]. Insulin is known to stimulate the production of nitric oxide as well as the production of ET-1 in endothelial cells [24-26]. In the healthy state there is a balance between the insulin stimulated release of NO and ET-1 from the endothelium. In individuals with IR or hyperinsulinemia the production of nitric oxide is decreased but the stimulatory effect of insulin in ET-1 is preserved leading to vasoconstriction and hypertension [27]. This could also explain the higher ET-1 values seen in the Icelandic horses compared to the Standardbred horses, as elevated insulin levels could cause an increase in ET-1 concentration [28]. The higher plasma ET-1 levels seen in the Icelandic horses in the present study did not cause an elevation of the systemic blood pressure.

In horses, cortisol production has a circadian rhythm with peak levels in the early morning and a nadir at night [12,29]. Because of the circadian rhythm sampling results are also influenced by sampling time [12]. Investigation of cortisol in untrained Standardbred horses in their home environment showed a circadian rhythm with a peak between 6 and 9 am [12]. These results suggest that the horses in the present study were sampled at the time of peak cortisol concentration. The circadian rhythm can easily be abolished by any disturbances like fasting and removal from the accustomed environment [12,13]. The Icelandic horses in the present study had significantly lower concentrations of plasma cortisol than the Standardbred horses. The concentrations measured in the Icelandic horses, are similar to the concentrations measured in Icelandic horses in an earlier study [30]. In that study, no differences in the concentrations of serum cortisol could be detected between Standardbred and Icelandic horses feed the same diet [30]. Our groups of horses were not feed the same diet, but previous research have failed to show any impact of different diets on cortisol concentrations in horses [29]. The mean concentration of cortisol of the sedentary Standardbred horses in the present study is in good agreement with the mean concentration previously reported in well trained cross-bred trekking horses indicating that the differences in cortisol concentrations was influenced by other factors than fitness status [31]. Chronic inflammation is known to depress cortisol concentrations in horses [32] and systemic inflammation is an important component of EMS [33]. The lower mean cortisol concentrations shown in the Icelandic horses in the present study could be related to breed differences, partial IR or be management dependent.

After transportation and exposure to a new environment, plasma ET-1 concentrations differed significantly from the concentrations measured in the home environment. This is likely related to a stress reaction caused by the transportation and the new environment. No significant differences were detected in the cortisol concentrations measured after transportation compared to the measurements in the home environment. This is in contrast to findings from a previous study, were higher serum cortisol levels were found in horses that had been transported compared to a group of non-transported horses [31]. The reason for this discrepancy in results between studies could be related to differences in sampling time. The horses in the present study spent a minimum of 12 hours in the new box stalls before the sample collection took place which might have been long enough for the pulsatile secretion of cortisol to reestablish [13,34]. In the study by Medica *et al.* (2010), the sample collection was carried out the same day as the transportation occurred and the time interval between transportation and sampling might have been too short for a reestablishment of the pulsatile secretion. The results of the present study indicate that serum cortisol and indirect blood pressure can be measured with reliable results if samples are collected at least 12 hours post-transportation. This is supported from findings from a study in donkeys were transportation altered the normal rhythm of resting cortisol to an increased rate of continuous secretion in both fed and fasted animals with a reestablishment of the circadian rhythm 8.5-10.5 hours later [13].

The significant difference in the concentrations of ET-1 and cortisol between the two breeds was not reflected in any differences in blood pressure. Previous studies have found that laminitis prone ponies have a different metabolic profile compared to non-laminitic ponies with significantly higher BCS, plasma insulin concentrations as well as decreased cortisol concentrations [4,35]. The metabolic profile and the predisposition for laminitis also seem to be inherited [35]. It is possible that the differences reported in plasma ET-1 and serum cortisol between breeds also are inherited and part of a metabolic profile associated with EMS. However, this must be further investigated on a larger material of horses of different breeds.

Both blood pressure measurement devices gave values that showed an acceptable interday variation. The indirect oscillometric blood pressure technique is known to overestimate the systolic blood pressure in dogs compared to the direct blood pressure technique [23-37]. However, if the clinician is aware of the possible risk that indirect blood pressure measurement techniques might overestimate the blood pressure, the risk of falsely diagnosing hypertension in horses appears to be little. In the present study, the HDO device gave higher values in systole compared to the Cardell device. This could partly be related to the difference in cuff size between the two devices. The size of the cuff is known to influence the obtained blood pressure values where a cuff that is too wide underestimates the blood pressure whereas a cuff that is too narrow tends to overestimate the values. The optimum cuff width for horses is one-fifth of the tail circumference [38]. It is therefore important that the cuff used is adjusted for each type of horse and the greater cuff size of the Cardell might be preferable in horses with a wide tail, such as some of the Icelandic horses in the present study. However, the results from the present study indicate that both devices can be used to measure systemic blood pressure in Standardbred as well as Icelandic horses. There are results that indicate that the HDO device has a better correlation with the direct blood pressure measurement technique compared to the Cardell device [39]. A previous study in dogs also showed that although the blood pressure values obtained with the HDO device showed a greater variability than the values obtained with the direct blood pressure measurement technique, there was a good correlation between the two techniques [37]. Both devices can be run on battery, which makes it possible to use them during field conditions. The results from this study showed that the Cardell device had less interday variation in systole compared to the HDO device. However, the Cardell is large and impractical to handle in a field setting whereas the HDO device is smaller in size, lighter and easier to handle. The previously reported high sensitivity and good correlation with the direct blood pressure measurement technique for the HDO device is also advantageous.

## Conclusions

In conclusion, indirect blood pressure measurements, plasma ET-1 and serum cortisol all showed an acceptable interday variation and are candidates for further investigation in horses with Equine metabolic syndrome. Both the Cardell and the HDO device can be used for measurement of indirect blood pressure in horses, but the HDO was most practical to handle, especially in a field setting. There seem to exist a breed difference in the concentrations of plasma ET-1 and serum cortisol. Transportation and housing in a new environment seemed to influence the concentrations of plasma ET-1 but not serum cortisol. All of these factors need to be considered when indirect blood pressure, plasma ET-1 and serum cortisol are used in a clinical setting. Future studies that investigate the possible seasonal variation in blood pressure, ET-1 and cortisol on healthy horses of different breeds and of horses clinically affected of EMS are desirable.

## Competing interests

The authors declare that they have no competing interests.

## Authors’ contributions

JBR and KNO took equal responsibility for designing the study as well as coordinating the project and apply for funding. The practical experiment was conducted with equal contribution from JS, JBR and KNO. JS drafted the manuscript and KN supervised the drafting. All authors commented on the manuscript. All authors read and approved the final manuscript.
